# Religious and Spiritual Dimensions of Pro-Ana Discourse on X: A Linguistic Analysis for Counseling Practice

**DOI:** 10.3390/bs15121626

**Published:** 2025-11-26

**Authors:** Krisy Elrod, Angeliki Trifonopoulos

**Affiliations:** Department of Counseling and Human Services, University of Scranton, Scranton, PA 18510, USA

**Keywords:** pro-anorexia, spirituality, religious language, eating disorders, computer-assisted text analysis, LIWC, counseling practice

## Abstract

Anorexia nervosa is among the most lethal psychiatric conditions. Online pro-anorexia (“pro-ana”) communities may frame starvation and restriction in moral or spiritual terms. This study explored how pro-ana discourse on X (formerly Twitter) encodes values, spirituality, and identity through language, with attention to clinical practice. A dataset of 2396 English-language tweets (2020–2025) was collected using dual criteria (pro-ana hashtags plus eating-disorder keywords). Only U.S.-based English tweets were included to maintain linguistic and cultural coherence with LIWC-22 norms and counseling frameworks developed in U.S. contexts. Tweets were separated into three corpora (full, hashtags, and tweet bodies) and analyzed using Linguistic Inquiry and Word Count 2022 (LIWC-22), supplemented with custom spirituality and pro-ana dictionaries, and keyword/keyness analysis against a 36-billion-token web reference corpus. Religious language appeared consistently higher in hashtags compared with tweets and Twitter norms. Tweets contained more authenticity and self-disclosure, while hashtags functioned as collective markers of identity and practice. Body and food terms were strongly elevated, and affiliation terms appeared comparatively suppressed. Keyness analysis identified distinctive items such as *prayer fast*, *fasting prayer (Luke)*, *OMAD fast*, *hunger hurt*, and *I’m punching*, illustrating how sacred, cultural, and diet-related slogans were combined within pro-ana discourse. Pro-ana rhetoric may function as a sacralized identity frame that can provide existential meaning to disordered practices. These findings contribute to behavioral science by highlighting how online communities linguistically construct health-related identities and values. They also suggest that effective clinical interventions should address eating disorders not only at behavioral and cognitive levels but also at the level of values and spirituality.

## 1. Introduction

Anorexia nervosa ranks as one of the deadliest psychiatric disorders, with mortality rates higher than many other serious mental illnesses (Arcelus et al., 2011; American Psychiatric Association, 2022). Beyond issues related to weight or appearance, anorexia nervosa can also involve moral and spiritual perspectives. Online pro-anorexia nervosa (“pro-ana”) communities are loose digital networks of individuals who valorize restrictive eating as a means of control, belonging, and transcendence. Members often describe starvation as a disciplined or even sacred vocation that offers meaning and mastery over the body. These motivations of control, identity, and perceived purity anchor the moral tone of pro-ana discourse. Hashtags, slogans, and metaphors can linguistically elevate these practices into shared rituals of belonging (Chancellor et al., 2016; Yeshua-Katz, 2015; Lerman et al., 2023).

Professional organizations across mental health disciplines emphasize competence in understanding clients’ spiritual and religious expressions, including the competencies developed by the Association for Spiritual, Ethical, and Religious Values in Counseling (ASERVIC, 2009) and the American Psychological Association (Vieten et al., 2016). Pro-ana discourse presents a challenge for these skills, as sacred values are intertwined with disordered practices. When hunger is seen as prayer or fasting as purification, practitioners must pay attention to both the genuine feelings expressed in the language and the unhealthy strategies through which these feelings are acted out. Spiritually integrated psychotherapy offers one approach to this task by supporting sacred meanings without reinforcing harmful behaviors (Pargament, 2007; Vieten et al., 2016). This finding resonates with evidence that spirituality is often central to the lived experience of women with eating disorders, regardless of religious affiliation, underscoring the importance of integrating sacred dimensions into treatment (Akrawi et al., 2015).

Pro-ana rhetoric has shifted from early internet forums to algorithm-driven platforms like Twitter, TikTok, and Instagram, where hashtags such as #thinspo, #bonespo, and #anaismyreligion serve both as identity markers and rhetorical tools. Zappavigna (2015) describes hashtags as tools of “ambient affiliation,” fostering community through shared values. In pro-ana groups, hashtags can sanctify disorder by embedding starvation into collective slogans. Recent studies show that this content spreads widely and can quickly influence body dissatisfaction among viewers (Branley & Covey, 2017; Cobb, 2016). New research indicates that exposure to pro-anorexia TikTok videos can significantly lower body image satisfaction and increase internalization of beauty standards after just minutes of viewing (Blackburn & Hogg, 2024). This blurring of personal self-disclosure (tweet text) and communal identity performance (hashtags) complicates both assessment and intervention, requiring clinicians to distinguish lived suffering from broader sacred narratives circulating in these communities.

Several theoretical perspectives help clarify these dynamics. Moral Foundations Theory (Haidt & Joseph, 2004) suggests that human moral reasoning is rooted in intuitive domains such as care, fairness, loyalty, authority, liberty, and sanctity/purity. Pro-ana discourse often relies on themes of sanctity and purity, where eating is framed as contamination, fasting as cleansing, and hunger as a sign of strength or spiritual discipline. This rhetoric echoes ascetic traditions, in which bodily denial is seen as a way to achieve purification or transcendence (Rampling, 1985; Stammers, 2020). Spiritual bypass (Cashwell et al., 2007) further explains this pattern, where psychological distress is wrapped in sacred metaphor, allowing embodied pain to be seen as spiritual progress. In this way, starvation can be linguistically reframed as devotion, with suffering hidden beneath sanctified discourse.

Self-Determination Theory (Ryan & Deci, 2000) offers an additional interpretive perspective. The basic psychological needs of autonomy, competence, and relatedness may be linguistically reframed in this context: autonomy as strict self-denial, competence as mastery over hunger, and relatedness as a sense of belonging mediated through sacralized hashtags rather than through genuine relational exchange. These distortions align with research linking anorexia nervosa to perfectionism and cognitive rigidity (Steinglass et al., 2006) and demonstrate how values themselves, not just symptoms, can be diverted in maladaptive ways.

Methodologically, computer-assisted text analysis (CATA) provides systematic tools for exploring these rhetorical features. Linguistic Inquiry and Word Count 2022 (LIWC-22) measures the proportion of words within validated psychological categories such as religion, authenticity, analytic thinking, and social affiliation. It also produces summary variables that index stylistic dimensions (Pennebaker et al., 2022). LIWC has been used to analyze online mental health discussions to uncover the values behind digital communities. Extending this method to pro-ana discourse enables an empirical examination of how anorexia may be linguistically sacralized and how this differs from general Twitter norms. Complementing LIWC, custom dictionaries (spirituality and pro-ana-specific terms) and keyness analysis (G^2^ and log ratio against a 36-billion-token reference corpus) offer converging evidence to strengthen interpretive rigor.

The present study systematically explores the religious, spiritual, motivational, and stylistic aspects of pro-ana discourse on Twitter. By comparing pro-ana tweets and hashtags with LIWC Twitter norms and analyzing distinctive keywords and phrases, the study clarifies how sacralized values are formed linguistically. The findings are presented within a framework that supports clinical practice, emphasizing spiritually integrated care and alignment with professional competencies.

Three research questions guided this study:

1. How frequently, and in what forms, does religious and spiritual language appear in pro-ana discourse?
2. How does the motivational and stylistic profile of pro-ana tweets and hashtags differ from LIWC Twitter norms?
3. What implications do these linguistic patterns hold for spiritually integrated and values-informed clinical practice?

## 2. Materials and Methods

### 2.1. Design and Ethical Safeguards

This study used a cross-sectional, observational design with computer-assisted text analysis (CATA) to explore the moral, spiritual, and motivational language of pro-anorexia (“pro-ana”) discussions on X (formerly Twitter). The analysis combined three approaches: (a) Linguistic Inquiry and Word Count 2022 (LIWC-22), (b) custom dictionaries related to spirituality and pro-ana-specific terms, and (c) keyword and keyness analysis (G^2^ and log ratio). Following recommendations for CATA in counseling research (e.g., Ni et al., 2025), multiple methods were employed to improve triangulation and confidence in interpretation.

The study protocol was reviewed and deemed exempt by the authors’ Institutional Review Board. All procedures adhered to ethical guidelines for internet-based research, including minimizing participant risk and analyzing only publicly available data. To protect anonymity, exemplar tweets reported in the manuscript are paraphrased composites that preserve rhetorical features without reproducing verbatim user content.

### 2.2. Data Collection

Tweets were collected through the X API v2 (Pro Tier) and custom Python 3.13.7 scripts between January 2020 and January 2025. Search criteria required each tweet to contain both (a) a recognized pro-ana hashtag (e.g., #proana, #thinspo, #bonespo, #fasting, #anaismyreligion) and (b) an eating disorder–related keyword (e.g., restrict, fast, goal weight). This dual requirement ensured that the dataset reflected both explicit community affiliation and anorexia-related discourse.

Inclusion criteria required tweets to (a) be written in English, (b) originate from U.S.-based accounts, and (c) valorize pro-ana practices. Exclusion criteria removed retweets, advertisements, verified accounts, advocacy posts, and recovery-oriented content. After filtering, the final corpus included 2396 tweets. Tweets were restricted to U.S.-based, English-language accounts to align with the cultural context of ASERVIC competencies and the LIWC-22 normative database, ensuring interpretive consistency across moral and spiritual vocabulary. 

### 2.3. Preprocessing

Standard text preprocessing steps were performed. All text was converted to lowercase, and usernames, URLs, emojis, and extraneous punctuation were removed. Tokens representing meaningful values (e.g., calorie counts, fasting durations) were retained. Hashtags were separated from tweet content so each could be analyzed both separately and together. This resulted in three distinct corpora: (1) full text (tweets plus hashtags combined), (2) hashtags only, and (3) tweet bodies only.

To allow flexibility in downstream analyses, each corpus was further processed into versions with and without stopwords and into single-token and multiword-token formats. This setup supported comparisons across different levels of granularity (such as unigram versus bigram or trigram) and ensured that both isolated words and recurring phrases could be identified.

### 2.4. Linguistic Measures

The cleaned corpora were analyzed with LIWC-22 (Pennebaker et al., 2022). LIWC determines the percentage of words in a text that belong to psychologically validated categories. Consistent with CATA best practices, the unit of analysis was the aggregate corpus rather than individual tweets. This approach was chosen to capture the collective rhetorical profile of pro-ana discourse.

Given that LIWC norms derive from longer texts, comparisons here are approximate. Tweets were analyzed one per line so that LIWC treated each as a single case, aligning with LIWC’s normative database. However, tweets are shorter than many texts in the LIWC reference corpus, and this length difference may influence comparisons. Results are therefore presented descriptively, with log ratio effect sizes used to indicate the direction and magnitude of divergence rather than precise benchmarking.

Six “star categories” were selected a priori as most relevant to counseling and values discourse: Religion, Authenticity, Analytic Thinking, Power, Achievement, and Affiliation. Four supporting categories were also included for clinical context: Anxiety, Body/Physical, Ingest/Food, and Death. LIWC summary variables (Analytic Thinking, Clout, Authenticity, Tone) were extracted for descriptive purposes. Prior research has demonstrated LIWC’s capacity to distinguish between pro-anorexic and recovery communities, supporting its use in this context (Lyons et al., 2006).

### 2.5. Custom Dictionaries

Two custom dictionaries were developed to supplement LIWC:

1. Spirituality dictionary: captured sacred, purifying, or ascetic language absent from LIWC’s Religion category but common in pro-ana discourse (e.g., ascetic, cleanse, ritual).
2. Pro-Ana dictionary: captured community jargon, shorthand, and identity markers (e.g., ana, restrict, bones, gw).

In this study, religious language referred to explicit institutional or doctrinal expressions (e.g., prayer, God, church), whereas spiritual language denoted experiential or existential references to purity, transcendence, or sacred identity that were not tied to institutional faith. These operational distinctions guided the development of the spirituality dictionary and ensured that the analysis captured both formal and nontraditional forms of sacralized language. 

Dictionaries were developed iteratively. The two authors independently compiled candidate terms based on prior research, disciplinary expertise, and pilot tweet samples. A subject-matter expert in religion reviewed the lists, suggested additions, and rated their conceptual alignment. Overlapping terms with existing LIWC categories were removed to prevent double-counting. Inter-rater agreement for inclusion reached 95%. Selected terms are shown in the text; complete dictionaries are available upon request.

In addition, a small set of applied lexical categories was created to capture constructs frequently observed in pro-ana discourse but not represented in LIWC (e.g., “Methods_Behaviors,” “Weight_Goals,” “Body_Image,” “Community_Meta”). These categories were used as a validity check to ensure that content characteristic of the community appeared in expected contexts (e.g., hashtags signaling identity, tweets containing body- and authenticity-related talk).

### 2.6. Keyword and Keyness Analysis

Keyword analysis was used to identify distinctive lexical items beyond LIWC categories. Keyword frequencies in the pro-ana corpora were compared with reference frequencies from the enTenTen20 web corpus (~36 billion tokens). The enTenTen20 corpus was chosen because it represents a large, contemporary, web-based collection of English-language texts, providing a robust baseline for detecting linguistic distinctiveness related to general online communication. Analyses followed Sketch Engine conventions, combining G^2^ and log ratio to identify statistically and substantively distinctive words and phrases.

The study corpus contained approximately 61,600 tokens. Relative frequencies were calculated for each candidate item, and log ratios (log_2_ of the study’s relative frequency divided by the reference frequency) were used to measure distinctiveness. Since tokenization created overlapping variants (e.g., *fasting prayer Luke*, *days fasting prayer Luke*), results were manually deduplicated, keeping the most representative forms. The final table included 11 unique items covering religious slogans (*prayer fast*, *fasting prayer [Luke]*), diet culture phrases (*low cal snack*, *OMAD fast, unused receptors*), embodied experiences (*hunger hurt*), and cultural borrowings (*I’m punching*).

## 3. Results

### 3.1. Overview of Analytic Sequence

The analysis proceeded in three stages: (a) LIWC-22 comparisons between pro-ana corpora (tweets, hashtags, and full corpus) and LIWC Twitter norms, (b) custom dictionary checks for pro-ana-specific and spirituality-related language, and (c) keyword/keyness analysis of distinctive lexical items against a 36-billion-token reference corpus. Reporting follows a corpus-level aggregation approach, with findings triangulated across methods to highlight broad rhetorical patterns supported by concrete linguistic examples.

### 3.2. LIWC “Star” Categories

Six categories were identified a priori as most relevant to counseling and values discourse. Religion, Authenticity, Analytic Thinking, Power, Achievement, and Affiliation showed distinct contrasts with Twitter norms.

Religion. Religious language was markedly elevated in hashtags (5.93%, log ratio +3.46) relative to tweets (0.93%, +0.78) and LIWC Twitter norms (0.54%). This suggests that sacralized rhetoric (e.g., *prayer*, *fasting*, *blessed*) was concentrated in hashtags functioning as community slogans.

Authenticity. Tweets contained substantially more authenticity/self-disclosive language (67.85%, +0.37) than hashtags (1.00%, −5.71). Hashtags rarely conveyed personal disclosure; instead, they served as collective markers of identity.

Analytic Thinking. Both tweets (66.61%, +0.64) and hashtags (89.00%, +1.05) exceeded Twitter norms (42.86%). Hashtags were primarily analytic, reflecting their role as condensed, structured signals of meaning. Tweets were less analytic but still above baseline, blending narrative disclosure with structured rhetoric.

Power and Achievement. These categories remained near normative levels. Hashtags showed a slight elevation in power (1.84%, +0.53) and slight suppression in achievement (0.93%, −0.39). Tweets were nearly identical to norms (power 1.16%, achievement 1.23%).

Affiliation. Affiliation terms were lower than norms across corpora (tweets 1.73%, −1.31; hashtags 1.22%, −1.81; full 1.65%, −1.38). Although belonging was central to community identity, it was more often implied through hashtags than expressed through explicit affiliative words (e.g., *family*, *together*).

Table 1 presents percentages and log ratios for all six star categories.

### 3.3. LIWC Supporting Categories

Four supporting categories (Anxiety, Body/Physical, Ingest/Food, and Death) provided additional clinical and contextual insight.

Anxiety appeared modestly lower than norms across corpora (tweets 0.10 vs. 0.16).

Body/Physical terms were sharply elevated (tweets 6.86 vs. 2.17; hashtags 14.70).

Ingest/Food terms were also elevated (tweets 3.71 vs. 0.61; hashtags 4.74).

Death language approximated normative levels (tweets 0.14 vs. 0.17; hashtags 0.46). Table 2 presents these findings.

### 3.4. Custom Dictionary Checks

Custom dictionaries confirmed that pro-ana content appeared in expected contexts. Hashtags disproportionately contained pro-ana identity markers and practice slogans, whereas tweets contained higher levels of body-related and authenticity language. These results corroborate the LIWC patterns, reinforcing the interpretation that hashtags serve as communal identity markers while tweets capture lived experience.

### 3.5. Keyword and Keyness Analysis

Keyness analysis identified distinctive words and phrases with disproportionately high frequency relative to the enTenTen20 reference corpus. After deduplication, 11 unique items captured the rhetorical signature of pro-ana discourse.

Religious slogans include prayer fast, fasting prayer (Luke), fraternity, family, friends, ministry, and an anchored pro-ana identity in sacralized language.

Diet slogans: diet level, low cal high protein, low cal snack, low cal low carb, OMAD fast, unused receptors reflected calorie restriction and fasting practices.

Embodied experience: *hunger hurt* conveyed the physical sensation of starvation while echoing cultural references (e.g., Fiona Apple’s lyric in *Paper Bag*).

Cultural borrowing: *I’m punching* (dating slang repurposed as aspirational language) illustrated how external idioms were reframed in the service of pro-ana identity.

Table 3 presents the final deduplicated list of keywords and their log ratios.

### 3.6. Integration Across Methods

Taken together, the three analytic approaches present a clear picture. LIWC results show increased religion and analytic thinking in hashtags, greater authenticity in tweets, reduced affiliation, and strong focus on body and food language across datasets. Custom dictionaries confirm the expected presence of pro-ana and spirituality-related content, while keyness analysis provides specific examples of slogans and phrases that shape the discourse. This triangulated evidence boosts confidence that pro-ana hashtags serve as collective signals of sacralized discipline, while tweet bodies reflect more personal and embodied disclosures.

## 4. Discussion

This study identified several distinctive features of pro-ana discourse on X (formerly Twitter), including the sacralization of restriction, divergence between hashtags and tweet text, and distortions of basic psychological needs. Together, these findings demonstrate how pro-ana communication functions not only as symptom talk but also as a system of meaning laden with values that complicates both behavioral science research and clinical practice.

### 4.1. Sacralization of Restriction

A key finding is that pro-ana discourse goes beyond describing disordered behaviors to create values-based meanings that sacralize restriction. Religious language was especially common in hashtags, which serve as ritual-like slogans framing fasting and bodily denial as acts of devotion. Phrases like *prayer fast* and *fasting prayer (Luke)* directly connect restriction to biblical practices, while *fraternity family friends ministry* embeds pro-ana identity within campus ministry settings, highlighting intersections between faith, youth culture, and eating disorder risk (Chancellor et al., 2016; Yeshua-Katz, 2015; Lerman et al., 2023). Previous research also shows that pro-ana communities can act as algorithmically reinforced echo chambers, amplifying extreme identity claims and shielding them from challenge (Lerman et al., 2023). From a clinical view, this pattern reflects spiritual bypass (Cashwell et al., 2007), where psychological suffering is reframed as sacred commitment. Expressions like *hunger hurt* illustrate this process, naming the physical pain of starvation while reinterpreting it as purification. Thus, embodied distress is reframed in sanctified terms, which may sustain harmful practices and make disclosure more difficult.

### 4.2. Divergence Between Hashtags and Tweets

Another important finding is the difference between hashtags and tweet text. Tweets scored higher on authenticity and included more first-person disclosures, while hashtags were more analytical and filled with religious or practice-oriented terms. This indicates that online discourse functions on two levels. Tweets reflect individual experiences, whereas hashtags express collective identity through ritualized slogans (Zappavigna, 2015; Cobb, 2016).

This divergence has both theoretical and practical implications. From a clinical standpoint, it emphasizes how identity formation online is shared through personal stories and community symbols. Tweets can reveal clients’ internal thoughts, while hashtags indicate the larger sacred narrative that frames those struggles. Paying attention to both levels, personal expression and community identity, is crucial for understanding how meaning is shaped in pro-ana communities.

### 4.3. Distortion of Psychological Needs and Values

The findings also show how basic psychological needs described in Self-Determination Theory (Ryan & Deci, 2000) may be expressed through language. Autonomy was shown as strict self-denial, competence as control over hunger, and relatedness as belonging conveyed through sacralized hashtags rather than clear affiliative language. Although LIWC affiliation scores were lower than norms, a sense of belonging appeared indirectly through collective religious slogans and identity markers.

The keyword analysis confirmed these distortions. Diet slogans (*low cal snack*, *OMAD fast*, *unused receptors*), religious phrases (*prayer fast*, *fasting prayer [Luke]*), and cultural borrowings (*I’m punching*) show how discursive strategies from nutrition culture, spirituality, and popular slang come together to support pro-ana identities. These findings indicate that values like discipline, purity, and connection are present but are linguistically redirected in unhealthy ways.

These linguistic patterns also reflect the egosyntonicity of anorexia nervosa, or the extent to which disordered behaviors and beliefs are experienced as congruent with one’s values and identity. Many pro-ana expressions frame restriction as moral virtue, aligning self-control with self-worth. This egosyntonic framing helps explain why spiritualized and moralized language can maintain illness persistence despite physical decline (Serpell et al., 2002; Treasure & Schmidt, 2013). Within counseling, recognizing this alignment between moral identity and symptom expression is essential for developing interventions that decouple worth and virtue from restriction. 

### 4.4. Clinical and Behavioral Science Implications

Taken together, these findings highlight the importance of addressing eating disorders through values and meaning-making, not just behaviors. When clients describe fasting as prayer or hunger as sacred endurance, practitioners are encouraged to recognize the underlying yearnings while disentangling them from harmful practices. Spiritually integrated psychotherapy provides tools for affirming sacred commitments while guiding them toward life-affirming actions (Pargament, 2007; Vieten et al., 2016). Acceptance and Commitment Therapy can assist clients in seeing religiously charged self-talk as thoughts rather than absolute truths (Hayes et al., 2011), while narrative therapy offers opportunities to reframe sacred identity claims around compassion, relational belonging, and embodied dignity.

Professional competencies underpin this responsibility. Both the ASERVIC (2009) spiritual and religious competencies and the APA’s guidelines for psychologists (Vieten et al., 2016) emphasize the importance of differentiating adaptive from maladaptive spirituality, addressing spiritual bypassing, and helping clients reframe values into recovery-aligned virtues. The study shows how online communities linguistically construct health-related identities. The use of LIWC, custom dictionaries, and keyness analysis demonstrates how computational methods can uncover the moral and spiritual aspects of digital discourse. By decoding sacralized language, practitioners can gain a better understanding of how cultural, religious, and motivational values are co-opted to support disordered practices.

For practitioners, these findings underscore the need to integrate values clarification and sacred language awareness into assessment and treatment planning. Counselors can explore clients’ use of religious or spiritual metaphors, such as fasting as prayer or purity as moral strength, to uncover the deeper meanings attached to restriction. Interventions may include values clarification, narrative reconstruction of sacred metaphors, and psychoeducation, distinguishing adaptive from maladaptive spirituality. Preventively, clinicians and educators can collaborate with campus ministries, digital literacy programs, and faith leaders to counteract the moral glorification of starvation. Such interdisciplinary efforts extend beyond symptom reduction toward reshaping sacred identity around compassion, embodiment, and relational belonging.

## 5. Limitations and Future Research

Several limitations should be considered when interpreting these findings.

The dataset was drawn exclusively from publicly available, English-language tweets on X (formerly Twitter). Pro-ana discourse also circulates on platforms such as Instagram, TikTok, Reddit, and Tumblr, where imagery, videos, and algorithmic amplification strongly influence community meanings. By focusing only on textual tweets, this study may underrepresent multimodal elements that are central to the aesthetic and emotional appeal of pro-ana communities. Future research should employ cross-platform, multimodal approaches that integrate text, images, and videos to account for the entire ecosystem of eating disorder discourse.

LIWC-22 is a validated and widely used tool for quantifying psychologically meaningful categories, but as a dictionary-based method, it cannot capture contextual nuance such as irony, metaphor, or polysemy. For example, the term *cleanse* may denote dietary restriction, emotional renewal, or spiritual purification. Computational approaches have been widely used to model large-scale social factors in individual mental health, demonstrating the value of applying text analysis to online discourse (De Choudhury, 2025).

Dictionary approaches often risk reducing complex meanings into simple categories (Pennebaker et al., 2022). In this study, combining custom dictionaries with keyness analysis helped address this issue. Nonetheless, future research should merge computational methods with qualitative discourse analysis or ethnographic approaches to uncover layered meanings.

Tweets were analyzed one per line so that LIWC treated each tweet as a single case, aligning with LIWC’s normative database. However, tweets are shorter than many texts used to establish LIWC norms. As a result, standardized differences and log ratios should be viewed as approximate indicators of magnitude rather than precise effect sizes. Developing field-specific baselines for eating disorder discourse, or dividing corpora into pseudo-texts that resemble normative lengths, would improve interpretive validity.

This study offers a snapshot of discourse from January 2020 to January 2025. Pro-ana communities are flexible, changing in response to cultural events, moderation policies, and recovery efforts. For instance, the COVID-19 pandemic impacted social media use and eating habits, which may influence the discourse documented here. Long-term studies could reveal shifts in rhetoric over time, including the appearance of ambivalence, resistance, or recovery-focused discussions.

Although sacralized language is prominent, this design cannot determine whether such language reflects deeply internalized beliefs or performative participation in community norms. Some users may present fasting as a genuine spiritual practice, while others may use religious hashtags mainly as identity markers. This distinction is important for both researchers and clinicians: future studies could combine computational analysis with interviews, surveys, or ethnographic methods to better understand how online rhetoric relates to lived experience.

This study aggregated tweets at the corpus level and therefore did not account for individual differences among users. Variables such as illness stage, identity salience, or engagement level could influence linguistic style. Future studies incorporating author-level metadata or mixed effects modeling could identify within-user variance and clarify how personal characteristics shape pro-ana rhetoric. 

Keyword comparisons relied on the enTenTen20 web corpus (~36 billion tokens) as a reference standard. While comprehensive, this corpus reflects general online English rather than health- or counseling-specific discourse. Some terms may therefore seem artificially distinctive due to the corpus composition. Developing field-specific benchmarks, for example, comparing pro-ana corpora with pro-recovery or neutral health communities, would offer more context-aware interpretations. For example, research on recovery-focused online groups demonstrates how values and meaning can be redirected toward adaptive ends, offering a useful contrast to pro-ana discourse (Popoola, 2024).

Although the findings have clear counseling implications, they should be generalized with caution. Not all individuals who use pro-ana rhetoric online will bring such narratives into therapy, and those who do may differ systematically from more active community members. Future research should explore how online discourse appears in counseling sessions, including whether hashtags and slogans show up in dialogue and how practitioners respond in real time.

These limitations highlight several promising directions. Cross-platform, multimodal studies could more effectively capture the ecology of pro-ana discourse. Mixed-methods approaches combining computational text analysis with qualitative exploration would deepen interpretive insights. Corpus segmentation or field-specific benchmarks would enable more accurate normative comparisons. Longitudinal research could observe how sacralized discourse changes across cultural shifts. Lastly, client-centered triangulation linking online discourse with interviews or session transcripts would clarify how digital rhetoric relates to lived experience and clinical practice.

## 6. Conclusions

This study indicates that pro-ana discourse on X (formerly Twitter) is not just neutral communication about eating disorder behaviors, but a linguistically constructed identity framework where values, spirituality, and sacrality are central. Compared to Twitter norms, pro-ana discourse exhibits several unique features: religious language is mainly found in hashtags, authenticity appears more frequently in tweets, and body/food terms are consistently emphasized, while affiliation terms are relatively rare. Distinctive slogans like *prayer fast*, *fasting prayer (Luke)*, *OMAD fast, hunger hurt*, and *I’m punching* demonstrate how sacred language, diet culture, embodied pain, and cultural borrowings come together to shape pro-ana identity.

Taken together, these findings suggest that starvation can be linguistically framed as a vocation, reinforced through ritualized hashtags and collective identity. For clinicians, this study emphasizes how online discourse can transform health-related practices into identity systems filled with sacred and moral significance. For practitioners, it highlights that eating disorders cannot be fully understood or effectively addressed without considering the values and meaning systems they are part of.

Effective treatment, therefore, requires more than just symptom reduction or cognitive restructuring. Clinicians are encouraged to address the level of values, distinguishing genuine spiritual or existential yearnings from the harmful practices they are connected to. Spiritually integrated psychotherapy, Acceptance and Commitment Therapy, and narrative approaches all offer frameworks for affirming sacred commitments while guiding them toward recovery-aligned goals.

This study makes a modest but meaningful contribution by documenting sacralized language in pro-ana communities, clarifying the divergence between communal hashtags and personal tweets, and linking these linguistic features to counseling and behavioral science. Recovery, in this light, involves not only the reduction in disordered behaviors but also the reconstitution of moral and spiritual frameworks in ways that affirm life, connection, and dignity. Clinically, the findings highlight that pro-ana discourse intertwines faith, control, and belonging in ways that require spiritually informed, value-sensitive counseling interventions. Preventively, professionals can apply these insights to digital education, early identification of sacralized symptom talk, and collaborative outreach between clinicians and faith communities. 

## Figures and Tables

**Table 1:** Comparison of Pro-Ana Corpora with LIWC Twitter Norms: Star Categories.

Category	Tweets %	Hashtags %	Full %	Twitter Norms %	Log Ratio (Tweets)	Log Ratio (Hashtags)	Log Ratio (Full)
Religion	0.93	5.93	1.75	0.54	0.78	3.46	1.7
Authenticity	67.85	1	47.87	52.33	0.37	−5.71	−0.13
Analytic Thinking	66.61	89	71.19	42.86	0.64	1.05	0.73
Power	1.16	1.84	1.27	1.27	−0.13	0.53	0
Achievement	1.23	0.93	1.19	1.22	0.01	−0.39	−0.04
Affiliation	1.73	1.22	1.65	4.29	−1.31	−1.81	−1.38

Note. Values represent percentage of words in each corpus. Norms are from LIWC-22 Twitter reference data. Log ratios > 0 indicate higher rates in pro-ana discourse relative to norms; negative values indicate suppression.

**Table 2:** Comparison of Pro-Ana Corpora with LIWC Twitter Norms: Supporting Categories.

Category	Tweets %	Hashtags %	Full %	Twitter Norms %
Anxiety	0.1	0.17	0.11	0.16
Body/Physical	6.86	14.7	8.16	2.17
Ingest/Food	3.71	4.74	3.88	0.61
Death	0.14	0.46	0.19	0.17

Note. Supporting categories provide clinical context but were not selected as primary values-framing categories.

**Table 3:** Top Distinctive Keywords/Phrases in Pro-Ana Discourse Compared with Reference Corpus.

Keyword/Phrase	Study Frequency	Reference Frequency	Log Ratio
Diet level	90	0	25.66
Prayer fast	28	0	23.97
Fasting prayer (Luke)	24	0	23.75
Low cal high protein	20	0	23.49
Low cal snack	16	0	23.17
I’m punching	15	0	23.07
Hunger hurt	15	0	23.07
Low cal low carb	12	0	22.75
Turn on those unused receptors	12	0	22.75
OMAD fast (One Meal a Day slogan)	12	0	22.75
Fraternity family friends ministry	12	0	22.75

Note. These items derive from diet culture slogans and religious idioms, confirming LIWC-based trends.

## Data Availability

Due to the sensitive nature of pro-ana content, raw tweets cannot be shared publicly. However, keyword queries, custom dictionaries, and aggregated LIWC and keyness results will be made available on the Open Science Framework (OSF) [https://osf.io/n8t2d/?view_only=d53f88ca69cd4e4687bc402fc5f9e151; accessed on 28 February 2025]. Additional materials may be requested from the author.
